# Equity and Geography: The Case of Child Mortality in Papua New Guinea

**DOI:** 10.1371/journal.pone.0037861

**Published:** 2012-05-25

**Authors:** Anna E. Bauze, Linda N. Tran, Kim-Huong Nguyen, Sonja Firth, Eliana Jimenez-Soto, Laura Dwyer-Lindgren, Andrew Hodge, Alan D. Lopez

**Affiliations:** 1 Burnet Institute, Melbourne, Victoria, Australia; 2 School of Population Health, The University of Queensland, Brisbane, Queensland, Australia; 3 Institute for Health Metrics and Evaluation, University of Washington, Seattle, Washington, United States of America; Chancellor College, University of Malawi, Malawi

## Abstract

**Background:**

Recent assessments show continued decline in child mortality in Papua New Guinea (PNG), yet complete subnational analyses remain rare. This study aims to estimate under-five mortality in PNG at national and subnational levels to examine the importance of geographical inequities in health outcomes and track progress towards Millennium Development Goal (MDG) 4.

**Methodology:**

We performed retrospective data validation of the Demographic and Health Survey (DHS) 2006 using 2000 Census data, then applied advanced indirect methods to estimate under-five mortality rates between 1976 and 2000.

**Findings:**

The DHS 2006 was found to be unreliable. Hence we used the 2000 Census to estimate under-five mortality rates at national and subnational levels. During the period under study, PNG experienced a slow reduction in national under-five mortality from approximately 103 to 78 deaths per 1,000 live births. Subnational analyses revealed significant disparities between rural and urban populations as well as inter- and intra-regional variations. Some of the provinces that performed the best (worst) in terms of under-five mortality included the districts that performed worst (best), with district-level under-five mortality rates correlating strongly with poverty levels and access to services.

**Conclusions:**

The evidence from PNG demonstrates substantial within-province heterogeneity, suggesting that under-five mortality needs to be addressed at subnational levels. This is especially relevant in countries, like PNG, where responsibility for health services is devolved to provinces and districts. This study presents the first comprehensive estimates of under-five mortality at the district level for PNG. The results demonstrate that for countries that rely on few data sources even greater importance must be given to the quality of future population surveys and to the exploration of alternative options of birth and death surveillance.

## Introduction

Timely progress towards Millennium Development Goal 4 (MDG 4) continues to be a cause for concern in many developing countries. Between 1990 and 2015, MDG 4 targets a two-thirds reduction in under-five mortality, which is defined formally as the probability of death per 1,000 live births between birth and the age of five years. Previous studies suggest that less than a quarter of low-income and middle-income countries examined are on track to achieve this goal. [1]–[3] While there is compelling evidence to suggest that, worldwide, child mortality is declining, and, in fact, that this decline may be accelerating in several low-income sub-Saharan African countries, [3] cross-country and within-country inequalities in health outcomes still exist. [4]–[7] Although previous studies have explored disparities in child health using wealth quintiles, potential inequalities across geographical regions have been largely unexamined. [8]–[11] The studies in this area have tended to only examine a single geospatial dimension, often focusing on rural-urban disparities. [12]–[14]

In the case of Papua New Guinea (PNG), recent assessments show continued decline in the under-five mortality rate (U5MR) from approximately 100 per 1,000 live births in 1990 to 83 per 1,000 in 2010. [3] However, recognition of the challenges in meeting the MDGs within the 2015 time frame necessitated the adoption of country-specific goals, including the achievement of an U5MR of 72 per 1,000 live births by 2015. [15] PNG appears to be progressing towards this revised national MDG 4 target. Anecdotal evidence suggests that these national figures, however, mask regional inequalities and differences between rural and urban populations, yet comprehensive subnational analyses remain rare.

In many developing countries, such as PNG, birth and death registration systems are not yet sufficiently developed to allow accurate estimation of under-five mortality rates. Accordingly, methods have been developed to estimate under-five mortality from censuses and surveys. Previous analyses of inequities in mortality focused on reporting geographic distributions using population-based data sources, with the most comprehensive data available only at national and regional levels. In fact, the most complete under-five mortality analysis for PNG [16] was undertaken using 1980 Census data and was limited in its district-level estimation. However, the heterogeneity within PNG's provinces and the decentralised nature of the health system means that national- and regional-level data have limited value in planning and monitoring progress over time.

In this paper we report estimates of under-five mortality in PNG at national and subnational levels based on the most recent data available and advanced methods for calculating under-five mortality rates from summary birth histories. [17], [18] This is the first time that under-five mortality estimates at the district level have been produced for the entire country, which allows us to examine potential geographical disparities in child health. It is also the first time that the reliability of datasets available to measure child mortality has been formally assessed.

## Methods

### Ethics statement

The datasets used in this study were obtained from the Population and Social Statistics Division of the National Statistical Office of PNG, who was responsible for the management and review of the surveys, with technical assistance obtained from international consultants. Full review of this study from an institutional review board was not sought as the datasets were anonymous, with no identifiable information on the survey participants.

### Data

Three datasets from Papua New Guinea were identified as covering the time period of interest for the MDGs (i.e. 1990 to the present) and having the necessary modules to calculate under-five mortality rates: namely, the 1996 and 2006 Demographic and Health Surveys (DHSs) and the 2000 PNG Census. The datasets were cleaned by deleting duplicates and omitting data relating to children who had unfeasible birth dates and death ages (e.g. children reported to have died after the date of a survey interview).

There are two ways of collecting information about births and deaths of children from censuses or surveys that can then be used in statistical modelling. These are complete birth histories (CBHs) and summary birth histories (SBHs). The former permits direct estimation of under-five mortality rates, while the latter enables only indirect estimation. In surveys that include a complete history module, a mother is asked to report date of birth of all the children ever born and date of death of those who have died. Other information about each child, such as gender and birth order, is also collected. In surveys that only collect summary birth histories, a mother is asked to report the number of children ever born and the number of children that have died. We investigated the potential to compute both types of estimates for PNG. All statistical analyses described below were carried out using two statistical packages, Stata (version 10) and *R*.

### Under-five mortality estimates

Child mortality can be estimated directly from the CBHs reported in the two DHSs undertaken in PNG. These were nationally representative cluster sample surveys that covered 4,319 and 9,017 households in 1996 and 2006, respectively. Following the methods of Rajaratnam and colleagues, [17] we structured the CBH data from both surveys to detail the life or death in each month of a child's life from birth to the age of five years. Due to the relative rarity of deaths reported in such analyses, single-year estimates of under-five mortality are not sufficiently precise; hence estimates were made for every two-year period prior to the survey. Confidence intervals (CIs) on the direct estimates of under-five mortality were computed using standard simulation methods. [19]

In the absence of CBHs, under-five mortality rates can be computed from SBHs using newly developed methods [3], [17], [18]. We do this using the SBHs sourced from the 2000 Census. As described elsewhere, Rajaratnam and others' approach [17] incorporates two different methods, cohort-derived and period-derived, to compute under-five mortality indirectly. These methods use either the mother's age or the time since her first birth to create the estimates. Since the census did not include any questions on the time since first birth, we applied only the maternal age cohort-derived (MAC) method and the maternal age period-derived (MAP) method. In order to create a summary measure, we apply Loess regression to all the estimates generated from each of the two methods.

Several issues must be addressed to apply these indirect methods to PNG. First, for the MAP method, distributions of birth dates and death dates are created and applied to the total CEB and CD for each woman in each age group. This distributes all births and deaths to children of women in the respective age group over the past (up to) 25 years. The distributions are created to account for variation by geographic region and maternal grouping in 2-year groups. Papua New Guinea does not belong to any of the region groupings included in the model by Rajaratnam and others. Hence, we chose the distribution for ‘Sub-Saharan Africa, West and Central’, the region that most closely resembles PNG in terms of child mortality patterns.

Secondly, both the MAC and MAP methods run regressions that include country-level and region-level random effects. These effects were not estimated as part of the model developed by Rajaratnam and others since microdata were not available for Papua New Guinea. To address this, we applied the ‘Sub-Saharan Africa, West and Central’ Region's random effect in our regressions. All estimates take into account the sample weights that come with the data.

We capture uncertainty inherent in estimating under-five mortality from summary birth history information by accounting for parameter uncertainty in the summary birth history. This is achieved by simulating from the multivariate normal distribution described by the point estimates and variance-covariance matrices for the coefficients in each model. For 1,000 of these simulated sets of coefficients we estimate under-five mortality for each data point. Next Loess regression is performed to combine the MAP and MAC methods 1,000 times on each set of predictions produced using a simulated set of coefficients. We calculate the standard deviation of the 1,000 Loess series and then multiply this by two to reflect the fact that the standard error is artificially deflated by a factor of 0.5, given that we have used all the available data twice (i.e. once for MAC and once for MAP). Finally, we use this corrected standard error to estimate a 95% confidence interval, assuming a normal distribution.

### Procedure for testing the reliability of the survey data

In order to ensure the reliability of the under-five mortality estimates, we tested the survey data for its consistency. To test the reliability of the utilised survey data, we developed a procedure which is straightforward to perform and could easily be adapted to test the reliability of surveys in other countries. Our assessment took selected indicators from the DHSs and compared them to the census, with the assumption that the census represents the “population parameters”. Given that the only relevant census dataset available was the 2000 Census we could only assess the reliability of the DHS 2006. Both the census and the DHS 2006 have coincident indicators, such as questions on household assets. However, most of these indicators relate to the particular year that the census or the survey was conducted, which are often, as in our context, different. However, indicators related to age, such as age at the time of the survey or age of death, are mutable only by time. Hence, such indicators can be transformed onto comparable scales, assuming that the age structure does not change much between the census and the survey. Consequently, five-year age groups were chosen to preclude the problem of small sample sizes at individual ages. The following five indicators were used: (1) the proportion of men between the ages of 15 and 44 years who belong to each five-year age group; (2) the proportion of women between the ages of 15 to 44 years who belong to each five-year age group; (3) the average number of children ever born (CEB) in each five-year maternal age group; (4) the average number of children dead (CD) in each five-year maternal age group; and (5) the ratio of the total number of children dead to the total number of children ever born (CD/CEB) in each five-year maternal age group. This provided a total of 30 measures to test the reliability of the survey data. The census–derived estimates were assumed to represent the population parameters on two grounds: first, the census is a full survey of everyone in the country, and, secondly, as is common practice, the sample design for the DHS uses the previous census as the sampling frame.

Comparisons between the survey and the census data were made by verifying whether the confidence limits of the estimates computed from the DHS covered the population parameters as calculated by the census. Uncertainty in the birth months was introduced by creating numerous simulations through randomly selecting the birth month from a uniform distribution of feasible months. In situations where the confidence intervals did not contain the population parameter, the following two statistics were then calculated: (1) bias: the difference between the DHS estimate and the census estimate; and (2) per cent bias: bias divided by the true estimate from the census, multiplied by 100.

## Results

### Direct mortality estimates using DHS 1996 and DHS 2006

**Figure 1:**
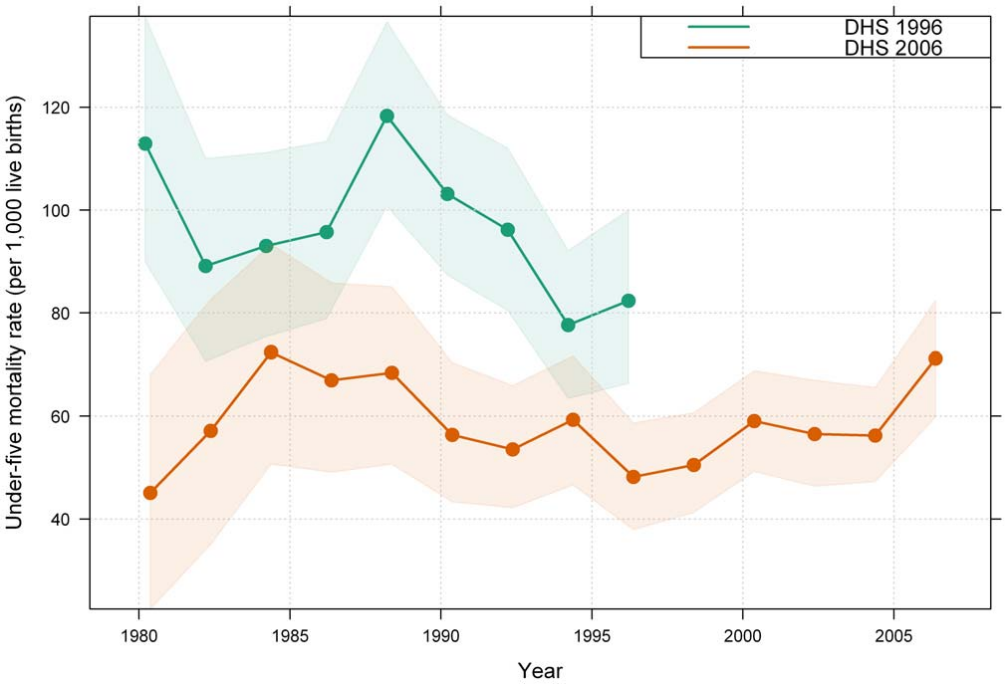
Bi-annual under-five mortality estimates and trends between 1980 and 2006 in Papua New Guinea. Data are from Demographic Health Surveys 1996 and 2006. Note: The solid line represents the under-five mortality estimates calculated in this study, while the shaded area represents the associated confidence intervals. DHS, Demographic Health Survey.

Figure 1 depicts the biannual trends in under-five mortality rates estimated separately using the DHS 1996 and DHS 2006 datasets. If the data were reliable we would expect the levels of under-five mortality computed separately from the two surveys to be fairly close to each other for the period over which the datasets overlap (i.e. 1980 to 1996). However, as Figure 1 shows, this is not the case. In the year 1990, for example, the estimated under-five mortality rate using the DHS 1996 was approximately 104 per 1,000 live births (95% CI: 89–120), whereas the estimate using the DHS 2006 was approximately 59 per 1,000 live births (95% CI: 45–73). In fact, on only four occasions do the confidence intervals overlap, and the overlap is not substantial. These results cast doubt over which, if either, of the datasets is accurate or reliable.

### Retrospective validation of DHS 2006

Given the discrepancy identified between the two surveys, we examined their reliability. The results of the data validation exercise are presented in Table 1. For thirty different 95 per cent confidence intervals, we expect to find approximately two (≈0.05×30) intervals that do not cover the population parameter. In fact, we found that over half of the measures derived from the DHS 2006 were biased, a figure much higher than could be attributed to chance alone. Our results show that the number of children ever born and the number of dead children were underreported, suggesting that the under-five mortality levels from the DHS 2006 are also underestimated. We also found that females surveyed in the DHS 2006 were not representative of the female population of Papua New Guinea. In particular, younger females were oversampled while older women were undersampled.

**Table 1:** Sampling bias of 2006 Demographic Health Survey, PNG.

	Census 2000			DHS 2006									
Type	Age group	N	Pop. para.	Age group	Sample size		Est.	Std. err.	95% CI		Covers “Pop. para.”?	Bias	% Bias
	2000			2006	no sw	sw			lower	upper			
Proportion of males	15–19	293,277	0.2365	20–24	1,718	1,658.5	0.2122	0.0054	0.2010	0.2222	no	−0.0243	−10.26
	20–24	239,863	0.1934	25–29	1,559	1,556.1	0.1991	0.0049	0.1894	0.2082	yes		
	25–29	219,680	0.1772	30–34	1,507	1,506.1	0.1927	0.0048	0.1825	0.2021	no	0.0156	8.80
	30–34	191,662	0.1546	35–39	1,210	1,220.8	0.1562	0.0046	0.1468	0.1649	yes		
	35–39	166,656	0.1344	40–44	1,020	1,016.9	0.1301	0.0043	0.1216	0.1383	yes		
	40–44	128,910	0.1040	45–49	816	857.3	0.1097	0.0043	0.1009	0.1177	yes		
Proportion of females	15–19	261,204	0.2168	20–24	1,917	1,879.2	0.2381	0.0054	0.2278	0.2490	no	0.0213	9.81
	20–24	234,938	0.1950	25–29	1,804	1,833.8	0.2324	0.0056	0.2216	0.2433	no	0.0373	19.14
	25–29	228,734	0.1899	30–34	1,494	1,501.4	0.1902	0.0051	0.1806	0.2005	yes		
	30–34	192,598	0.1599	35–39	1,213	1,217.0	0.1542	0.0046	0.1455	0.1636	yes		
	35–39	163,681	0.1359	40–44	894	880.3	0.1116	0.0040	0.1038	0.1197	no	−0.0243	−17.90
	40–44	123,458	0.1025	45–49	564	582.4	0.0738	0.0033	0.0675	0.0805	no	−0.0287	−27.98
Mean CEB by maternal age group	15–19	261,204	0.3989	20–24	1,917	1,879.2	0.2502	0.0165	0.2181	0.2841	no	−0.1488	−37.29
	20–24	234,938	1.2243	25–29	1,804	1,833.8	1.1639	0.0336	1.1012	1.2280	yes		
	25–29	228,734	2.2623	30–34	1,494	1,501.4	2.3041	0.0495	2.2081	2.4027	yes		
	30–34	192,598	3.3559	35–39	1,213	1,217.0	3.2245	0.0638	3.0994	3.3508	no	−0.1314	−3.91
	35–39	163,681	4.1006	40–44	894	880.3	4.2280	0.0925	4.0463	4.4166	yes		
	40–44	123,458	4.7407	45–49	564	582.4	4.7868	0.1160	4.5718	5.0190	yes		
Mean CD by maternal age group	15–19	261,204	0.0351	20–24	1,917	1,879.2	0.0188	0.0044	0.0100	0.0273	no	−0.0163	−46.52
	20–24	234,938	0.1006	25–29	1,804	1,833.8	0.0647	0.0078	0.0493	0.0802	no	−0.0359	−35.69
	25–29	228,734	0.1826	30–34	1,494	1,501.4	0.1501	0.0153	0.1187	0.1800	no	−0.0326	−17.83
	30–34	192,598	0.2764	35–39	1,213	1,217.0	0.2051	0.0187	0.1680	0.2401	no	−0.0713	−25.80
	35–39	163,681	0.3541	40–44	894	880.3	0.3120	0.0280	0.2558	0.3679	yes		
	40–44	123,458	0.4568	45–49	564	582.4	0.4333	0.0373	0.3566	0.5028	yes		
CD/CEB by maternal age group	15–19	261,204	0.0881	20–24	1,917	1,879.2	0.0751	0.0155	0.0449	0.1047	yes		
	20–24	234,938	0.0821	25–29	1,804	1,833.8	0.0556	0.0060	0.0429	0.0670	no	−0.0266	−32.34
	25–29	228,734	0.0807	30–34	1,494	1,501.4	0.0651	0.0060	0.0533	0.0763	no	−0.0156	−19.31
	30–34	192,598	0.0824	35–39	1,213	1,217.0	0.0636	0.0054	0.0526	0.0739	no	−0.0188	−22.77
	35–39	163,681	0.0864	40–44	894	880.3	0.0738	0.0060	0.0614	0.0851	no	−0.0126	−14.55
	40–44	123,458	0.0964	45–49	564	582.4	0.0906	0.0071	0.0762	0.1050	yes		

Note: For the DHS data, everyone was backdated to mid-July 2000, including children. DHS, Demographic Health Survey; N, number of observations; Pop. para., population parameter; est, estimate; std err, standard error; CI, confidence interval; sw, sample weight; CD, children dead; CEB, children ever born.

### Indirect mortality estimates using the 2000 Census

**Figure 2:**
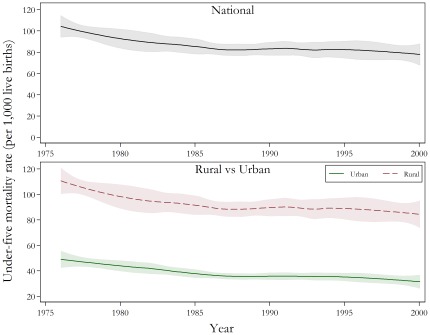
Under-five mortality rates for national, and rural and urban, populations between 1976 and 2000, PNG. Data is from the 2000 Papua New Guinean Census. Note: The solid line represents the under-five mortality estimates calculated using indirect methods, while the shaded area represents the associated confidence intervals.

While direct estimates from CBHs are generally preferable to indirect estimates from SBHs, the observed bias in the DHS 2006 data prevents their application in PNG. Figure 2 presents the estimated national under-five mortality rates between 1976 and 2000 with 95 per cent confidence intervals using indirect estimation from the 2000 Census. The average national U5MR reduced slightly from 104 deaths per 1,000 live births (95% CI: 94–114) in 1976, to 78 per 1,000 (95% CI: 67–87) in 2000. Mortality rates declined the most between 1977 and 1979, with an average rate of reduction of approximately three per cent per annum. In the decade 1990 to 2000, child mortality declined slowly, with a slight acceleration from 1997.

Beyond the national under-five mortality rates, Figure 2 also shows the sizeable disparity in rates between rural and urban areas. National estimates more closely follow rural mortality estimates since the vast majority of the population resides in rural areas. The rate of reduction in child mortality has been slower in rural PNG than urban areas. From 1976 to 2000, there were declines of around 1.07 per cent per annum in under-five mortality rates in rural areas versus 1.78 per cent per annum in urban areas.

**Figure 3:**
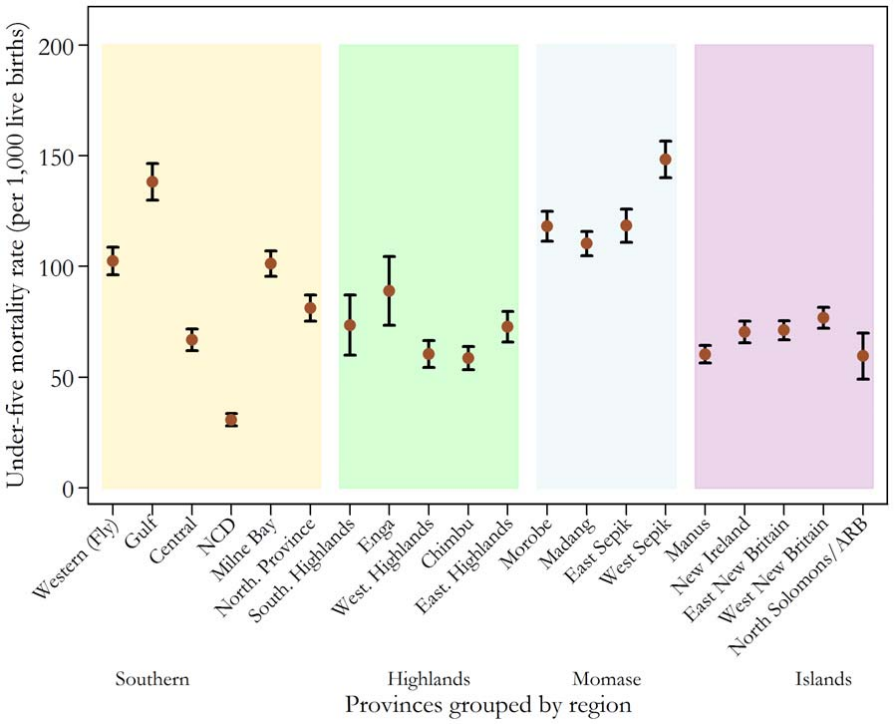
Estimated under-five mortality rates for PNG's provinces from 1976 to 2000. Data is from the 2000 Papua New Guinean Census. Note: NCD, National Capital District; ARB, Autonomous Region of Bougainville.

Further disaggregation of the data shows clear variation within and between PNG's four geographic regions. Based on the average regional estimates, the Momase (Northern) Region had the highest estimated U5MR (approximately 105 deaths per 1,000 live births), whilst the Islands Region had the lowest (approximately 72 deaths per 1,000 live births). Figure 3 presents the provincial averages with 95 per cent confidence intervals over the period 1976 to 2000. The most notable intraregional variation occurred in the Southern Region, which encompasses both strongly performing provinces, such as the National Capital District (NCD) and Central Province, as well as Gulf Province, which has the second-highest U5MR in the country. Conversely, the provinces within the Islands Region showed very little variation, with each achieving similar, comparatively moderate levels of child mortality.

**Figure 4:**
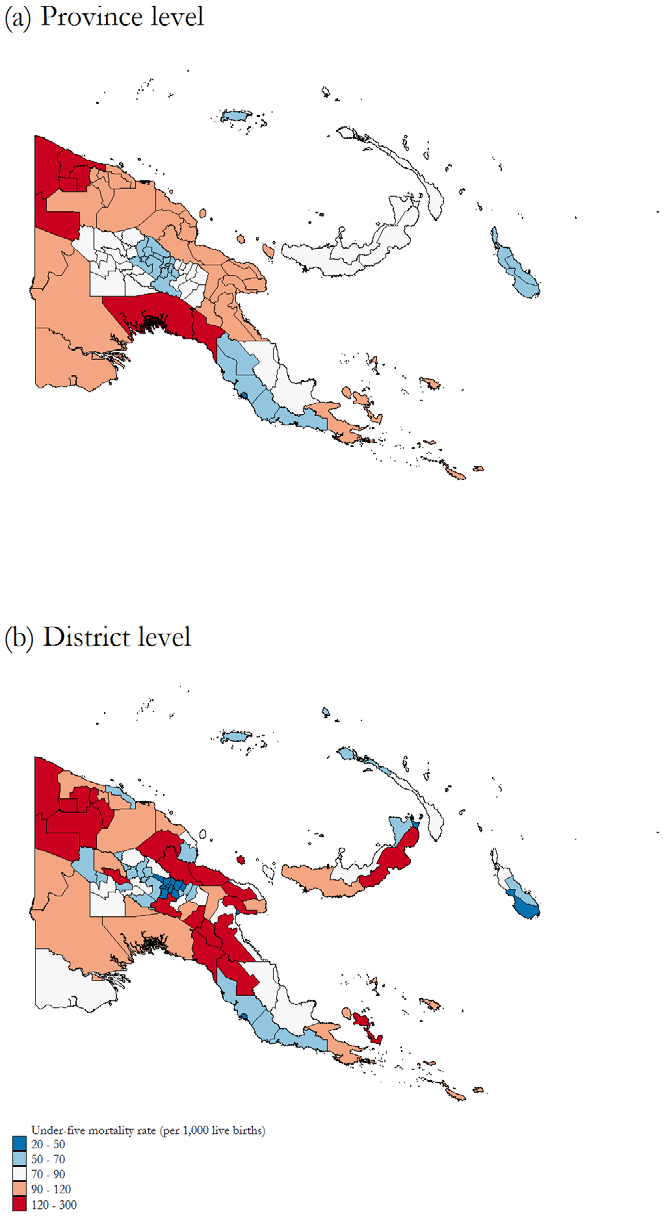
Under-five mortality rates at province level versus district level between 1976 and 2000, PNG. Data is from the 2000 Papua New Guinean Census. Note: source of the map is Global Administrative Areas (www.gadm.org).

The spatial distribution of under-five mortality across PNG is shown in Figure 4. Provinces and districts are grouped into categories according to estimated mortality rates. A comparison of U5MRs across districts reveals that neighbouring districts within a given province experience large differences in mortality. Some of the worst performing districts belong to the best performing provinces, and vice versa. For example, Karimui-Nomane, one of the three worst performing districts, belongs to Chimbu, one of the best performing provinces. In contrast, the district of Lae is in the top ten performing districts but belongs to Morobe, a poorly performing province. In both cases, the within-province disparity is associated with rural/urban characteristics. Karimui-Nomane is the most remote district in Chimbu, with very limited infrastructure and access, while Lae district covers the largest urban centre of PNG outside Port Moresby and is now a major commercial and industrial hub. Similarly, one can observe the large variation in the Eastern Highlands and Madang Provinces and the island of New Britain. We corroborate these observations by running basic one-way analysis of variance (ANOVA) tests for each year of the sample. This technique tests whether the province-level means and variances computed from the district estimates are equal across the 20 provinces. The results are presented in Table 2. Both sets of tests indicate that the means and variances of at least two provinces differ significantly. In the later years of the sample, the test-statistics for the equality of variance test have become larger; indicating that over time there is stronger evidence that the variation within provinces differs across PNG. The large variation within provinces reveals the value of district-level estimates.

**Table 2:** Within-province heterogeneity: Tests of equality of means and variances, PNG.

Year	Equal Means	Equal Variances
	F-stat.	χ^2^-stat.
1976	2.770	20.982
1977	2.772	22.025
1978	2.761	23.220
1979	2.744	24.421
1980	2.723	25.576
1981	2.696	26.620
1982	2.650	27.081
1983	2.602	27.263
1984	2.588	26.972
1985	2.571	26.479
1986	2.517	26.360
1987	2.430	26.543
1988	2.344	26.431
1989	2.249	26.493
1990	2.142	27.644
1991	2.009	30.829
1992	1.870	28.514
1993	1.760	27.397
1994	1.715	26.980
1995	1.716	27.576
1996	1.716	29.105
1997	1.713	32.695
1998	1.701	31.294
1999	1.669	31.547
2000	1.669	33.628

Note: The degrees of freedom for the F-test for equal means and the Bartlett's χ^2^ test for equal variances are (19, 67) and 17, respectively. stat., statistic.

**Figure 5:**
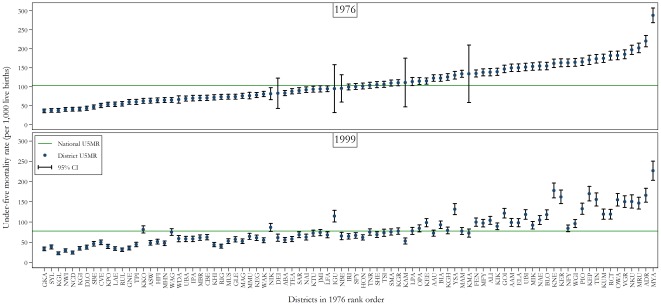
Under-five mortality rate estimates for all 87 districts of PNG in 1976 and 1999. Data is from the 2000 Papua New Guinean Census. Note: District codes and names are as follows: AAM, Angoram; AAU, Alotau; ABA, Abau; ADR, Ambunti-Drekikier; ALI, Aitape-Lumi; ASW, Anglimp-South Waghi; BIA, Bogia; BLO, Bulolo; CBE, Central Bougainville; CVE, Chuave; DEI, Dei; DLO, Daulo; ELA, Esa'ala; FEN, Finschafen; GKA, Goroka; GLE, Gazelle; GNE, Gumine; GOI, Goilala; HFI, Henganofi; HON, Huon; IGU, Imbonggu; IPA, Ialibu-Pangia; IRI, Ijivitari; JMI, Jimi; KAM, Kompiam; KEE, Kagua-Erave; KEG, Kavieng; KEP, Kandep; KER, Kerema; KGH, Kiriwina-Goodenough; KGI, Kerowagi; KGL, Kundiawa-Gembogl; KGR, Kandrian-Gloucester; KHI, Kairuku-Hiri; KIK, Kikor; KKO, Koroba-Kopiago; KMA, Komo-Magarima; KNE, Karimui-Nomane; KPO, Kokopo; KTU, Kainantu; KUM, Kabwum; LAE, Lae; LFA, Lufa; LPA, Lagiap-Porgera; MAG, Madang; MAM, Markham; MBR, Mul-Baiyer; MFY, Middle Fly; MHN, Mt Hagen; MIK, Maprik; MMU, Mendi-Munihu; MRU, Middle Ramu; MUS, Manus; MYA, Menyamya; NAE, Nawae; NAI, Namatanai; NBE, North Bougainville; NCD, National Capital District; NFY, North Fly; NIK, Nipa-Kutubu; NKU, Nuku; NWI, North Waghi; OPA, Okapa; OWA, Obura-Wonenara; PIO, Pomio; RCT, Rai Coast; RIG, Rigo; RUL, Rabaul; SAR, Sumkar; SBE, South Bougainville; SFY, South Fly; SHE, Sohe; SMA, Samarai-Murua; SYL, Sinasina Yongomugl; TEA, Talasea; TIN, Telefomin; TNR, Tambul-Nebilyer; TPI, Tari-Pori; TSI, Tawae Siasi; UBA, Unggai-Bena; UBI, Usino Bundi; VGR, Vanimo-Green River; WAG, Wabag; WAK, Wewak; WDA, Wapenamanda; WGI, Wosera-Gawi; YSA, Yangoru-Saussia. U5MR, under-five mortality rate; CI, confidence interval.

Figure 5 illustrates that, with a few exceptions, the overall rankings of the best and worst performing districts were fairly consistent across the 25-year period examined, suggesting that inequities between districts are entrenched in PNG.

Given the observed within-province variations in under-five mortality, geography does not appear to be the only driver of inequality. Hence we explored whether other variables could account for such variations by running a simple linear regression analysis for under-five mortality and poverty rates at the district level. As can be seen in Table 3, the results reveal a strong association between these two measures. A similar analysis reveals that districts with high mortality rates also have poor access to services. Although a lack of data prevented us from controlling for other variables, a multivariate regression analysis of mortality rates, with the inclusion of both poverty rate and access to services, suggests that both of these variables are strongly associated with mortality rates.

**Table 3:** Association between under-five mortality, poverty rate, and access to services between 1989 and 1999, PNG.

Dep. Var.: Under-five mortality	(1)	(2)	(3)
Poverty rate	1.605		1.059
	(0.281)		(0.299)
Access to services		−24.299	−17.587
		(3.675)	(4.081)
Constant	25.369	169.484	106.617
	(10.670)	(13.773)	(22.704)
*R^2^*	0.2327	0.2611	0.3425
*Adjusted R^2^*	0.2234	0.2522	0.3265
Observations	85	85	85

Note: Data are from 2000 Papua New Guinean Census and the World Bank's 2004 Papua New Guinea: Poverty Assessment Report. Heteroskedasticity-robust standard errors are reported in parentheses. Dep. Var., dependent variable.

## Discussion

This paper provides the first comprehensive measurement of under-five mortality in Papua New Guinea. Due to the lack of reliable data it is not possible to measure child mortality in the most recent decade (2000–10). The mortality analysis of the 2000 Census data suggests that, during the study period, PNG experienced a slow reduction in national U5MR at an average of 1.15 per cent per annum. If PNG does not experience changes that significantly accelerate the mortality reduction, achievement of the original MDG 4 target of two-thirds reduction in U5MR by 2015 will not be possible. However, the rate of change will be sufficient to meet the revised MDG 4 target adopted by the national government of 72 deaths per 1,000 live births. Importantly, subnational analyses reveal significant disparities between rural and urban populations, as well as inter- and intra-regional variations. The results suggest that the disadvantage experienced by rural populations is persistent, reflecting the fact that almost every socioeconomic indicator is significantly worse in rural areas. [20]

Significantly, the district-level estimates indicate that some of the best performing provinces have the worst performing districts, and vice versa. Such results indicate that inequalities in under-five mortality exist down to the district level; this reinforces the need for disaggregated measures of health outcomes. The within-province heterogeneity calls for caution when using aggregate estimates for planning and monitoring at subnational levels. This is especially relevant in countries which have decentralised health systems, such as PNG, where responsibility for health services has been devolved to provinces and districts.

Some consistency was found in the districts that performed well and those that performed poorly over the 25-year period examined, emphasising the entrenchment of inequities between districts in PNG. These districts were spread across the country, rather than concentrated in any one province. The district mortality differentials placed the mortality estimates for the best performing districts on a par with the estimates for central Asian countries, whereas the worst performing districts had under-five mortality estimates similar to, or worse than, countries in sub-Saharan Africa. [3]

The five consistently “best performing” districts had distinct advantages in the 1980s and 1990s in relation to access to services and transport thanks to a relatively high level of income. By comparison, the “poor performers” shared similar experiences, with little income from their own sources and limited support from provincial budgets, [20] as well as the challenges of very limited infrastructure, services, and governance capacity. However, even for the “best performing” provinces, the observed rates of under-five mortality decline were below the rate that would be required to achieve the unrevised MDG 4 target. Similarly, the equitable achievement of MDG 4 appears to be severely impaired unless highly accelerated rates of reduction have been achieved in the poorer performing locations during decade 2000 to 2010.

We found under-five mortality rates to be strongly correlated with poverty levels and access to services at the district level. Although the direction of causality is undetermined, one can argue that high poverty rates and low access to services and transportation are closely related. Indeed, a previous study [21] reported that, in PNG, poverty was related to low access to infrastructure and it proposed that the determinants of poverty were poor access to services, markets and transportation. This finding suggests that improving the levels of welfare and access to services in disadvantaged areas would contribute positively to PNG's progress towards the equitable achievement of MDG 4.

Analysis of data from the next census using the same method applied in this analysis is recommended. Such an exercise would allow the comparison and validation of the current findings and, most importantly, establish whether significant changes in under-five mortality levels between the two censuses exist. The limitations of the indirect estimation methods are well-documented in the literature. [17]–[18] In short, summary birth histories are subject to two main types of limitations. First, SBHs are used when CBHs cannot be estimated due to the lack of direct information on: (i) the number of recorded deaths that are child deaths; (ii) the location in time of births and (iii) the location in time of deaths. As a result, these parameters are inferred from indirect information taken from the patterns observed in the surveys. For example, one must create and apply regional distributions of births and deaths across time prior to the survey in the period-derived methods. Since these patterns are based on information from a wide variety of countries, the patterns are generalised across countries and across time. Through the application of local regression we seek to minimise this error. Secondly, since SBHs use survey-based data, some potential measurement error biases might be introduced. For example, only surviving women can be interviewed. Therefore, if child and mother survival are correlated there is the potential for the resulting mortality estimates to be biased. Moreover, SBHs use the age of the mother as an input for determining the time location of each estimate. In the presence of age-misreporting, estimates can thus be biased. Other well-known pitfalls of survey-based data may also create measurement error in summary birth histories. For example, the number of children born and children died can be incorrect due to misreporting or sampling error.

An unexpected finding was the difference in mortality trends estimated based on the DHS 1996 from those based on the DHS 2006 for the common period, that is, 1980 to 1996. When we studied this issue more systematically, the results of our validation exercise on the DHS 2006 as opposed to the 2000 Census identified substantial bias in the DHS 2006 sample that cannot be explained by chance alone. In general, there are three potential sources of bias: (1) the misreporting of birth dates for both mothers and children; (2) the omission of children; or (3) problems with the sampling of DHS 2006. In our data validation methodology we attempted to correct for, and reduce the effect of, the first two sources of bias by simulating every plausible birth date for both the mothers and children and by counting more children as dead. We concluded that the most likely cause of the biases is problems with the sampling of the DHS. Unfortunately, since we do not have access to the sampling documentation, we cannot identify the direct causes of such biases or the corresponding corrective measures. Furthermore, while our validation exercise focused on age-related indicators, it is likely that biases also exist in other indicators, especially those that belong to the Women Individual Questionnaire, such as maternal mortality and coverage of child health interventions.

The methodology proposed to test the reliability of the survey is easy to perform and could be adapted to test the reliability of surveys in other countries in a straightforward manner. However, researchers should bear in mind two important caveats related to the proposed method. First, the technique relies heavily on the quality of the dataset used as the standard to judge the reliability of the survey of interest. As outlined above, in our study the 2000 Census was a suitable candidate given that it covered the entire population and the sample design for the DHS uses the census as the sampling frame. A researcher would similarly need to provide some rationale for their choice of dataset. Secondly, our method utilised age indicators as the means of comparison. Consequently, we had to assume that the age structure did not change much between the year of the survey and the year of the census. It is possible that problems, such as age misreporting and recall bias, could be significant if the census and surveys are far apart in time. More generally, a researcher would have to ensure large population movements (such as international migration or internal displacement) do not occur in the period between the implementation of the survey and the implementation of the census. In the case of PNG, there was no evidence to suggest that such large population movements had occurred between 2000 and 2006. Researchers would similarly need to outline the assumptions employed depending on the indicators utilised for their particular country of interest.

Papua New Guinea's reliance on few data sources to measure progress in health and health equity puts even greater importance on the quality of those sources and their subsequent analysis. To usefully inform policy and planning decisions, data must be available at a level of disaggregation that complements the governance arrangements of the health system. Furthermore, improvements in both the quality of survey design and the management of data collection are necessary for future population surveys. Stronger national health information systems, along with alternative and feasible systems of birth and death surveillance, would contribute to Papua New Guinea's ability to effectively monitor and evaluate national health planning and progress towards reaching the Millennium Development Goals.
